# A Mouse Variable Gene Fragment Binds to DNA Independently of the BCR Context: A Possible Role for Immature B-Cell Repertoire Establishment

**DOI:** 10.1371/journal.pone.0072625

**Published:** 2013-09-02

**Authors:** Andrea Queiroz Maranhão, Maria Beatriz Walter Costa, Leonardo Guedes, Pedro Manoel Moraes-Vieira, Tainá Raiol, Marcelo Macedo Brigido

**Affiliations:** 1 Laboratório de Biologia Molecular, Instituto de Biologia, Universidade de Brasília, Brasília, Distrito Federal, Brazil; 2 Laboratório de Bioinformática, Instituto de Biologia, Universidade de Brasília, Brasília, Distrito Federal, Brazil; 3 Division of Endocrinology, Diabetes and Metabolism, Department of Medicine, Beth Israel Deaconess Medical Center and Harvard Medical School, Boston, Massachusetts, United States of America; Institut Jacques Monod - UMR 7592 CNRS - Université Paris Diderot, France

## Abstract

B-cell maturation occurs in several steps and requires constant stimulus for its continuing development. From the emergence of the pre-B-cell receptor, signal transduction stimulates and supports B-cell development. Current viewpoints indicate that both positive selection pressure for autoantigens and tonic signaling constitutively stimulate B-cell maturation. In this work, we tested for the presence of a putative DNA binding site in a variable gene segment in a germline configuration, independently of VDJ recombination. After a survey of the public antibody databases, we chose a single mouse heavy variable gene segment that is highly represented in anti-nucleic acid antibodies and tested it for ssDNA binding. A phage display approach was used to search for intrinsic binding to oligo deoxythymidine. The results revealed that binding to an antigen can be influenced by the use of a specific DNA binding V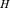 gene segment. Our data support the idea that some variable genes have intrinsic reactivity towards specific types of endogenous autoantigens, and this property may contribute to the establishment of the immature B-cell repertoire.

## Introduction

The adaptive immune system has evolved to become a highly efficient surveillance system. VDJ recombination was first introduced in the jawed animal lineage and is a major source of antigen receptor variability, allowing a multitude of B cell receptor (BCR) specificities in a polyclonal population that is constantly renewed from a pool of lymphocytes progenitors [1]. BCR-specific clonal expansion from a naive repertoire is an ancient and fundamental activity of adaptive immunity. The onset of clonal diversity with a broad repertoire of reactivities has been thoroughly examined, but the establishment of the naive repertoire is still less understood. In mice and humans, B cells are generated in the bone marrow and rely on the constant signaling of the bone marrow BCR [2]. This signaling may occur through antigenic stimulation from the nearby milieu [3]–[5] or antigenic-independent tonic signals [6], [7]. Although BCR signaling is fundamental for survival, strong signaling that is associated with self-antigen stimulation induces V gene edition or cell death, a quality control mechanism that prevents or reduces the chance of producing high affinity autoantibodies [8]–[10]. The resulting B-cells that leave the bone marrow produce the immature naive repertoire, which is further maturated in the periphery, yielding the circulating antibodies that protect and maintain homeostasis in the animal.

The antibody repertoire that leaves the bone marrow has been shown to be primarily auto- and polyreactive [11], apparently an outcome of positive selection for autoantigens in the early stages of receptor assembly [4]. Part of that autoreactivity is lost in the spleen and lymph nodes, where new reactivity is attained by V gene edition and somatic affinity maturation [8], [12]–[14]. Reactivity to DNA is part of the naive repertoire [11]. These naive anti-DNA antibodies are generally harmless, unless the cognate B cell clones progress to class switch and affinity maturation, leading to the production of pathological antibodies, a situation observed in autoimmune diseases such as systemic lupus erythematous [15]. Therefore, autoreactivity and cross-reactivity are the basis for an effective polyclonal response. They are the raw material for building high-affinity antibodies in germinal centers, where they suffer affinity maturation that is dependent on specific antigen accessibility. CD4 T cell and follicular dendritic cells assist in this maturation process [16], [17]. In this advanced stage, B-cell-producing antibodies mount a highly specialized and efficient protection against pathogens. Therefore, the initial repertoire is the key for an efficient B-cell response against antigens, and an intrinsic capacity of V genes for reacting to autoantigens could bias the naive repertoire towards an efficient and protective B-cell response. Intrinsic affinities for specific autoantigens are thought to be a selectable evolutionary trait.

The constant stimulation of BCR is a key factor in maintaining pre-B cell development [2]. Receptor signaling due to autoantigen stimulation along with a tonic basal signaling are reported to be necessary for pre-BCR formation and B-cell maturation [2]. Therefore, cross-linking antigens such as membrane-bound proteins and monotonous polymeric antigens may play an important role in initial BCR repertoire development [5]. As a consequence, it is plausible that the preservation of autoantigens€ reactive germline genes throughout evolution could have improved lineage specific B-cell survival. The preference for specific V genes can be observed for certain antigens [3], [18], and this preference leads to a progressive fixation of mildly reactive V gene segments. The current repertoire of V gene segments could be the result of constant selective pressure for gain or loss of biased V genes segments. Thus, a consequence of this hypothesis is that V gene segments that bias the binding of the B-cell receptor towards a ubiquitous multivalent antigen would be preserved during evolution due to their role as repertoire modelers. In this report, we address this hypothesis. Because anti-DNA polyreactive antibodies are widely observed in non-immunized humans and mice [11], germline reactivity to DNA may be one of these developmental modelers. We used phage display antibody HCDR3 (heavy chain complementary determining region 3) libraries to compare the selection profile of two mouse V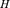 genes, one found to be frequently present in anti-nucleic acids antibody and another that has never been observed in such antibodies. If there is a V gene that is prone to bind DNA, the antibody that harbors it should be less dependent on CDR3 for binding. Deep sequencing analyses of phage display anti-ssDNA selected antibodies confirmed this hypothesis, suggesting that antigen-reactive germline V gene segment may have been selected during evolution.

## Results

### The V
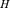
10 Family is Over-represented in Anti-DNA Antibodies

**Figure 1:**
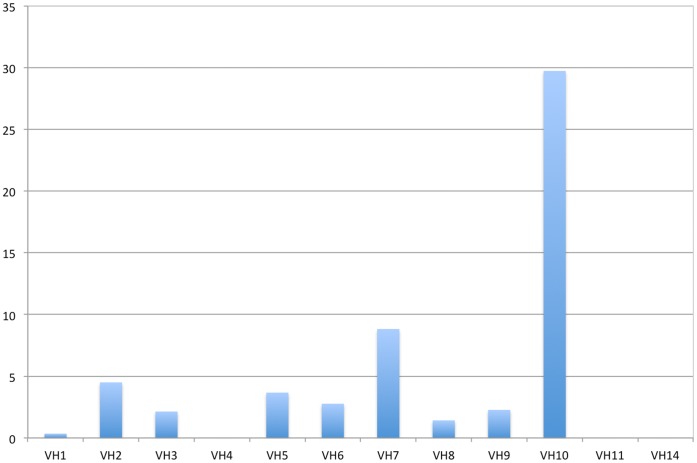
Representativeness of anti-DNA antibodies among mouse V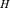 families. Red bars, the frequency of family-specific sequences in the database divided by the number of the family€s germline genes; blue bars, number of anti-DNA specific sequences divided by the total family’s sequence.

We analyzed two sequence databases, the IGMT, a dedicated database, and the IgBlast, retrieved from NCBI. A total of 11,986 mouse (*Mus musculus*) V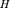 protein sequences were downloaded and classified following their closest germline using blastp. The distribution of the V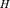 sequences is shown in Figure 1 and Table S1. Each V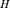 was also classified for its antibody specificity. A total of 3,548 sequences showed an “anti-” term and were classified as specific. Anti-nucleic acids and variations were filtered from this list and analyzed separately as described in the methods. From the initial 11,986 V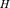 sequences, 750 were classified as anti-nucleic and 3,525 as not anti-nucleic. The classification of these sequences in families is shown in Table S1. Only V genes found at least 4 times were considered for analysis.

The V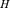 gene fragment usage was not shown to be uniform. Some families are found frequently, such as V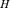 1 (J558) or V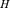 5 (7183), but others, such V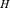 14 and V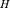 15, are rarely found. This finding may be partially explained by the differential number of germline sequences in each family. Some families are exceptionally large, such as the V
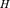
1 (J558) with 92 groups, but size does not completely explain the stochastic distribution among germlines (Table S1). V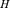 gene fragment usage in anti-DNA antibodies is also observed in almost every family, but some V gene families appear to have an exceptional rate of anti-DNA specificity (Figure 1). The V
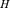
7 (S107) and V
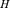
10 (DNA4) families appear preferentially in antibodies that bind to DNA. The V
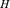
2 (Q52) and V
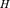
5 (7183) families also appear, to a lesser extent, to be overrepresented in anti-DNA antibodies. The usage of V
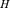
10 gene family members in anti-DNA antibodies is very high, given that 22 out of 37 are annotated as antibodies that bind to nucleic acids (Table S1). This suggests a bias for this V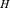 towards DNA binding. This family consists of only two V germline genes [19] and is poorly represented in the IMGT, but it is worth attention because more than half of the reported antibodies that use this V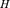 gene fragment are annotated as nucleic acid binders (Table S2). By contrast, the V
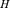
4 gene segment, which also appears rarely in the IMGT database, has not been reported in any anti-nucleic acid antibody.

### Intrinsic DNA Binding of the V
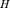
10 Gene Segment

Our hypothesis is that V
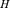
10 germline genes are prone to generate anti-DNA antibodies. Thus, a V
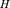
10 gene segment containing antibody should be less dependent on other gene segments that compose the antigen binding site for such a binding feature. Hence, a V
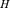
10-bearing Fv should be less dependent on both the HCDR3 and the light chain. An experimental system was developed based on the pCIg816 phage display vector [20], a phagemid created to obtain a scFv fragment fused to the M13 gene 8 on the bacteriophage capsid. In this vector, we introduced either a V
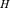
4 or a V
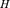
10 germline sequence along with a library of 9 mer HCDR3 and a JH4 gene fragment forming the variable heavy chain, concatenated with a fixed V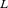 chain of the Z44 antibody [21]. This mAb binds to Z-DNA but not to ds or ssDNA. This V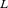 was used as a neutral element so that there would be no interference with direct DNA binding. In this context, the HCDR3 library component makes the final contribution to DNA binding, allowing the selection of anti-DNA scFvs distinguished only by their HCDR3. Two plasmids, pCIg 844 and pCIg 8410, were generated and harbored V
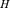
4 and V
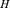
10 V gene germline segments, respectively. Both were used to receive the HCDR3 library (Figure S1).

A total of 45 clones of the V
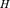
10 library and 53 clones from the V
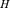
4 library were identified by Sanger sequencing after the second and third round of selection on oligo(dT). Two HCDR3s predominate among sequences, as they appears in most of clones of both the V
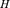
10 and V
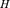
4 pools. In total, the V
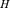
4 selection yielded three different sequences, while the V
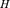
10 library yielded nine (Table 1).

**Table 1:** HCDR3 diversity in phage selection.

Round	Analyzed clones*^a^*		Unique HCDR3 sequences		Total sequence diversity*^b^*	
	V 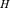 4	V 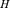 10	V 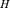 4	V 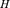 10	V 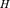 4	V 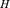 10
2	33	24	2	4		
3	20	21	3	9	3	9

Phage clones from the second and third selection rounds were analyzed using Sanger sequence.

A total of nine different sequences were obtained, three of then appears in V
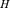
4 and V
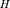
10 clones.

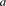
Sequence clones with accepted HCDR3 format were considered.

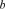
Unique sequences considering both rounds. Second round sequences were contained within third round sequences.

**Figure 2:**
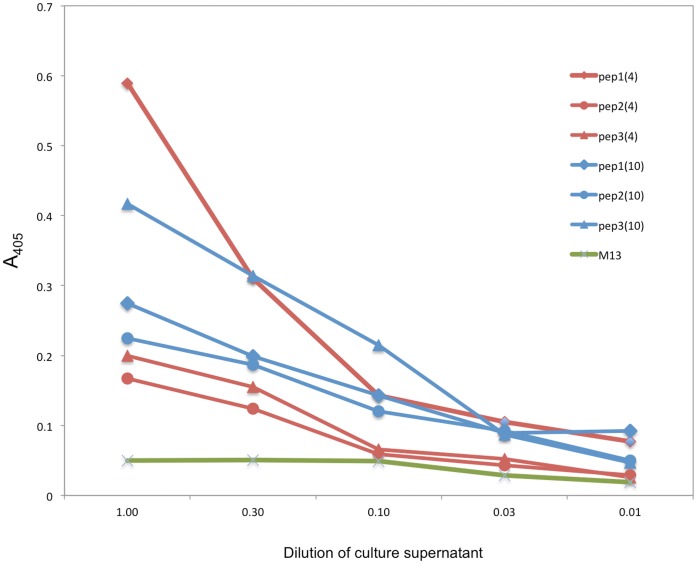
Binding of HCDR3 phage clones to ssDNA. Individual phage particles derived from chosen V
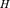
4 and V
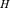
10 pools were tested. Phage clones with HCDR3 peptides in the context of V
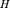
4 and V
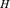
10 were chosen. Phage supernatant was transferred to oligo-dT adsorbed microplates and serially diluted. Phages were detected with anti-M13 antibodies. Blue, V
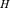
4 phage clones; red, V
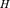
10 clones. Symbols differentiate HCDR3: diamonds, YLLSPLLLA, circle, VQQVNNLA e triangle VQYVNNALA. Green line represents the VCM13 helper phage as the negative binding control.

Clones isolated from V
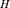
4 and V
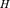
10 libraries selected on oligo(dT) were tested for binding in phage ELISA. Nine phage supernatant from the V
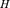
10 library and three from the V
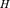
4 library were tested on oligo(dT) adsorbed microplates. A control M13 phage supernatant was used. Most recombinant phages bound to oligo(dT), and only three out of 12 clones showed a low binding profile comparable to the M13 control. Although all the V
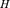
4 clones bound, the best binder was a V
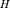
10 clone containing the peptide YLLSPLLLA in the HCDR3, the peptide that was most frequently found in the analyzed HCDR3. Interestingly, the V
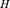
4 clone bearing this same peptide in the HCDR3 binds to oligo(dT), but to a lesser extent. In contrast, the peptide VQYVNNALA in the context of V
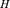
4 binds better than in the context of V
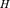
10 (Figure 2).

### Deep Sequencing of Phage Libraries

The Sanger analysis of individual phage clones revealed anti-DNA antibodies from both libraries, but the dominance of a few number of clones hampers any analysis of the universe of possible anti-DNA antibodies that can be select by this phage display system. Therefore, we proceeded with a deep sequencing analysis of these libraries using the Illumina platform. Phage pools after the third round of selection, as well as the original library (round zero), were sequenced. Primers specific for the V
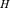
10 and V
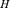
4 gene fragment were designed, including a bar code to differentiate samples according V genes and rounds. Table 2 summarizes the sequencing results, and valid HCDR3s were considered for further analyses. We found 78,019,444 sequences in R1 and 63,850,748 in the complementary sequence file R2. Unique sequences counts were found much less often: a total of 38,637 (22,733 translated peptides) for V
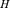
4 round 0; 22,811 (12,459 peptides) for V
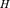
4 round three; 91.385 (35,778 peptides) for V
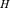
10 round 0; and 94.610 (36,162 peptides) for V
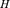
10 round three (Table 3). As observed for the Sanger sequencing of individual phage clones, two HCDR3 sequences, YLLSPLLLA and VQQVNNALA, dominated the libraries, encompassing 96.7% of all coded HCDR3. Interestingly, they did not come from particularly expanded clones but, rather, were coded by several nucleotide sequences (synonymous codons). Importantly, the nucleotide diversity was somewhat neutral and comparable in both libraries (Figure S2). Their representativeness also changed during selection, in opposite directions. YLLSPLLLA was positively selected in V
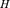
10 clones, while VQQVNNALA was positively selected in V
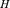
4 clones (Figure S3).

**Table 2:** Classified sequences for each phage pool.

Phage pool	Number of sequences for sequencingchannel per group		Frequence of sequencesper group (%)	
	R1	R2	R1	R2
V 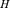 4 round 0	12,307,946	9,696,207	14.78	14.28
V 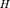 4 round 3	7,833,979	6,229,236	9.40	9.18
V 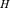 10 round 0	26,759,708	22,120,439	32.35	32.58
V 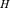 10 round 3	31,117,811	25,804,866	37.35	38.01
Not classified	5,283,947	4,040,576	6.34	5.95
Number of valid sequences	78,019,444	63,850,748		

Classified sequences after quality filtering and HCDR3 match.

**Table 3:** Unique sequences in each phage pool.

Phage pool	R1 - Total sequences	R2 - Total sequences	Uniques to R2*^a^*	Total nucleotide sequences*^b^*	Total peptide sequences*^c^*
V 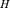 4 round 0	34,839	10,084	3,798	38,637	22,733
V 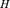 4 round 3	21,157	4,547	1,654	22,811	12,459
V 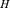 10 round 0	87,527	13,624	3,858	91,385	35,778
V 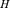 10 round 3	90,233	15,964	4,377	94,610	36,162

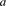
Number of unique sequences in R2 not identified in R1.

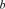
Total number of unique nucleotide sequences in each phage pool.

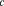
Total number of unique peptide sequences in each phage pool.

**Figure 3:**
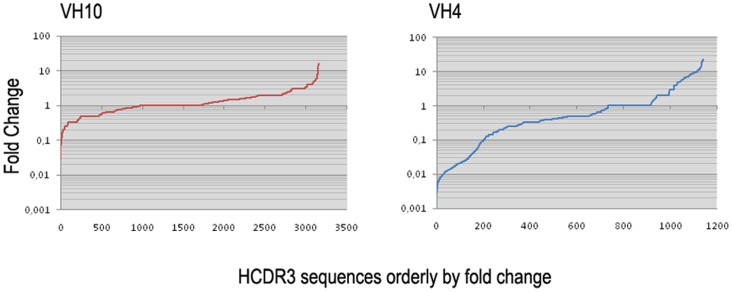
Individual HCDR3 peptide sequence Fold Change after round three of selection. The ratio of third to zero round counts was computed for each HCDR3 peptide. The Fold Change list was orderly and plotted sequentially for V
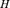
10 (right) or V
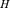
4 (left) libraries. Fold Change is shown in log scale.

The fold change for each HCDR3 peptide has been plotted by order (Figure 3). Both the V
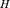
4 and V
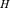
10 libraries had a large number of peptides that were selected positively (fold change
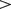
1) and negatively (fold change
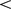
1) compared to their round zero phage pool. This result is consistent with a selection process imposed by oligo(dT). Considering only peptides with an improved fold change (over 4 fold in round three compared to round zero), there were similar numbers of V
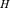
10 (137) and V
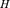
4 (124) sequences. These sequences represent positively selected HCDR3 clones. The oligo(dT) selection in the V
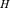
4 library resulted in the depletion of 342 sequences, considering a fold change lower than 0.25, contrasting with the 84 clones that were counter-selected for the V
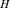
10 library.

**Figure 4:**
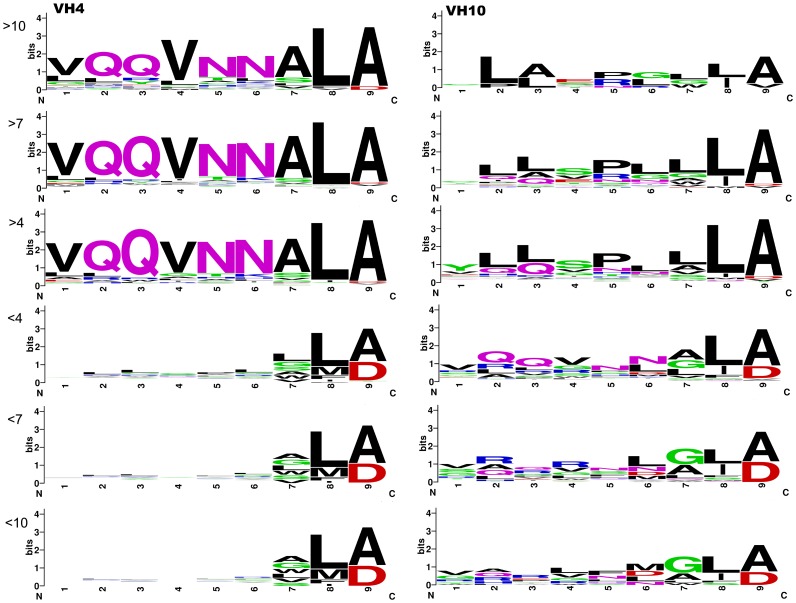
Information content of selected phage pools. Selection fold changes were divided into strata, and the information content in each stratus is quantified for every residue using Weblogo. The size of the residue reflects a bias toward its presence in that specific position at specific strata. Positively selected V
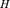
4 clones showed a large bias for the VQQVNNLA sequence, while negatively selected V
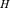
4 clones display no clear bias for any HCDR3 sequence.

HCDR3 clones selected or counter-selected may contain structural features that improve or abolish DNA binding. Therefore, we divided these positively and negatively selected pools into three groups: highly selected (fold change 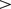
10 or 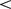
0.1), moderately selected (fold change 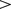
7 or 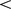
0.143), and mildly selected (fold change 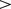
4 or 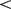
0.25). These stratified groups were analyzed for their information content using the Weblogo platform. Comparing V
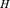
4 and V
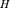
10 selection, we observed a strong enrichment of HCDR3 using peptides similar to VQQVNNALA for V
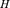
4, and under much less pressure, V
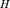
10 sequences converged to XLAXPLLLA (Figure 4). It is worth noting that to compute entropy, peptides were not weighted by their frequency, and the change in sequence profile reflected a collective change in HCDR3 diversity. Thus, VQQVNNALA was both the most frequent HCDR3 and the consensus among the highly selected V
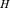
4 clones, reflecting the enrichment of HCDR3 towards this motif. Counter-selected HCDR3 sequence entropy suggests that there is no strong bias and no clear sequence consensus, especially for V
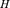
4 HCDR3, as observed based on the low information content of the first six residues (Figure 4).

**Figure 5:**
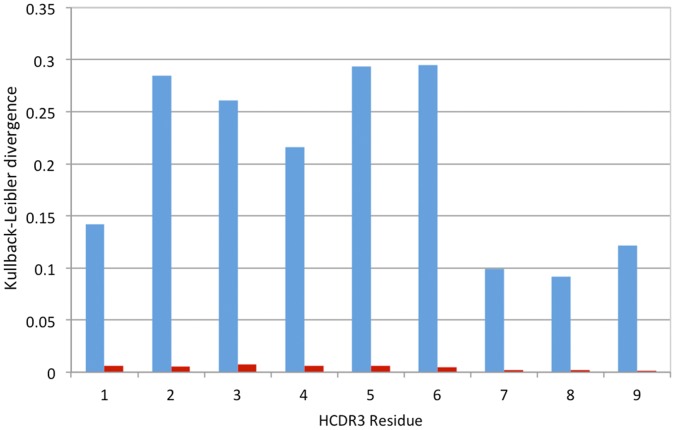
Kullback-Leibler distance of HDR3 pools. This measurement reflects the change in information content between distributions (before selection and after it) reflecting a selection bias. Entropy change from round zero to round three for each residue position is shown. Blue bars represent V
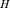
4 library HCDR3s, and red bars represents the V
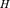
10 library.

To measure the information content change during the selection process, we calculated the Kullback-Leibler distance between round zero and round three for each library. In Figure 5, the change in information entropy is plotted for each HCDR3 residue. It is clear from the histogram that the selection on oligo(dT) imposes a higher change in entropy for the V
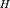
4 library, reflecting an increased prominent selective pressure and a more restrictive universe of selected HCDR3 (Figure 5).

## Discussion

The biological role of anti-DNA antibodies had been debated for a long time. Such antibodies may be part of a basic recognition set [16], helping maintain homeostasis by clearing dead cell content [22]. Alternatively, they may have appeared by chance, a consequence of BCR polyreactivity [4]. Because they can be detrimental, as evidenced by their correlation with certain pathologies [15], their preservation can be understood as a phenotypic trade-off. In this context, the presence of DNA binding-prone V genes would suggest a biologically relevant function, selected throughout the evolution of the immune system. The preservation of these genes may reflect a role in naive B-cell repertoire assembly [23]. Our data mining of mouse antibody databases revealed that some V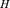 sequences are more frequently found in anti-DNA antibodies, while other sequences are never found. A single family is particularly over-represented in anti-DNA antibodies: the heavy chain gene segment fragment V
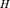
10. This family is not widely found in reported antibodies, but they are of note because more than half are described as anti-DNA antibodies. If these V gene family members harbor an intrinsic affinity towards DNA, it would suggest that the anti-DNA phenotype is encoded in the germline genome of the mouse. In this work, we tested whether a single V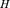 gene segment possesses an intrinsic affinity for DNA, indicating a conserved and potentially adaptive genetic trait.

We tested whether the use of V
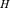
10 gene segments bias towards the formation of a DNA-binding BCR. Since antibodies are complex molecules, coded by a combination of many gene segments, testing for the contribution of individual V gene segments is a difficult task. The V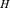 gene segment is part of the variable heavy chain domain of BCR. Thus, it is never produced alone in nature. Depending on the CDR3 and framework 4 of the heavy chain that folds along with a V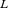 into a stable heterodimeric structure at the tip of the BCR (or its cognate soluble antibody). Therefore, we designed an scFv phage display experiment to test for V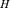-specific DNA binding by minimizing the effect of the remaining molecule. We produced an scFv fused to M13 gene 8, which contains a fixed V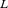 found in the anti Z-DNA antibody Z44 [21]. The choice of this V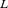 reflects an intention to provide a neutral background, reducing a possible affinity bias of the Fv. On the other hand, the choice of an anti-DNA V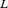 would facilitate the occurrence of an anti-DNA binding site. To evaluate the bias of V
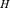
10 for anti-DNA activity, we compared it to a V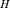 gene fragment that has never been described in an anti-DNA antibody. Like V
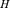
10, V
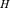
4 is a rarely used, small V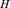 family. Thus, it seemed appropriate for the comparison. To reconstitute a functional V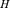 domain, we fused both a germinal V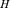 gene segment to a nine-residue HCDR3 library and the C-terminal framework 4 that completes the V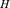 domain. Since the only difference between the HCDR3 libraries is the germline V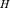, the selected phage clones reflect an intrinsic binding capability of V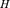 as a result of HCDR3 diversity observed in these clones. Consequently, a “permissive” V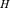 would allow a greater variety of HCDR3 in opposition to a “non-permissive” V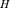 gene, which would necessarily rely on a restricted set of unique HCDR3s that would compensate for the lack of a wild-type antigen bias.

A phage display experiment was conducted to enrich the phage pool for ssDNA binding fusion phages after three rounds of selection. The use of a gene 8-based phage display system improves avidity and increases the number of anti-DNA phage clones [24]. Surprisingly, the second and third round was dominated by a few HCDR3 sequences. These dominant CDR3-derived phage clones were shown to bind DNA by ELISA, but their high frequency is likely to be a characteristic of the phage display system, considering their dominant presence in the original libraries (round zero) of both V
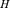
10 and V
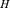
4. It is possible that primer diversity was more restricted than expected (Figure S2) or simply that these HCDR3 sequences better accommodate the introduction of a foreign gene in the bacteriophage capsid, improving phage assembly, infectivity or replication [25]. Therefore, to overcome the dominant effect of these few sequences, we performed deep sequencing of the original and the third round pool of both V
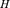
10 and V
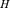
4 libraries. Among millions of HCDR3 sequences, over-represented HCDR3 peptides embody as much as 96% of the total set. The entire set of sequences was the focus of our investigation, and we considered only the sequence fold change and the information content (entropy) of libraries. Our search focused on the HCDR3 clones that were both positively and negatively selected during the selection cycles and the loss of variability during the selection process.

The observed population of HCDR3 clones revealed a marked loss of diversity in the V
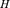
4 HCDR3 library compared to the V
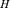
10 counterpart (Figure 5), suggesting that only a small set of HCDR3s is compatible with DNA binding in V
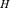
4-bearing clones. This observation supports the original hypothesis that an intrinsic affinity of the V
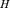
10 gene fragment towards binding to DNA would lead to less restriction of the possible HCDR3 universe. Moreover, searching for HCDR3 sequences that are enriched or depleted may help to understand the type of contribution a specific CDR3 can make to binding. It is interesting to note that the fold change enrichment between the library and select pools flats at 22-fold, which contrasts with a greater negative fold change in the counter-selected clones (Figure 3). It is conceivable to argue that gene 8 display improves the avidity of recombinant phage particles and may partially mask a real affinity gain. Interestingly, V
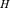
4 library clones exhibited a larger set of counter-selected clones, corroborating the hypothesis that V
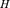
4 is not permissive for DNA binding and that only a restricted set of clones is positively selected after panning.

The analysis of the crystal structure of a V
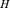
10 derived anti-DNA antibody also supports the germline intrinsic affinity of V
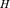
10 towards DNA. Comparing the primary sequence of both the V
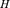
10 and V
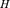
4 sequences reveals a highly divergent region in the HCDR2. This region was show to be fundamental for DNA binding in the BV04-01 model [26]. Two contiguous phosphate groups contact three V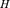 residues, two of them are encoded in V
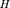
10 germline sequence (Figure S4), suggesting that germline sequences may also be able to bind ssDNA. The presence of a cationic pocket in the V
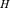
10 antibody may also explain the observed enrichment for asparagine-containing HCDR3s in V
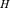
4 clones that is not observed in the mostly aliphatic HCDR3s bearing V
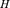
10 clones. V
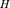
4 clones may require a more cationic HCDR3 to achieve anti-DNA binding to compensate the germline V
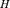
4 anionic HCDR2. Interestingly this HCDR2 binding pocket is found in another anti-DNA-favored family, S107 (data not shown). This HCDR2 region, which includes residues H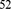-H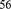, was also shown to be important for nuclear antigen binding [23]. This motif was shown to have a low anionic content of anti-nuclear antibodies, and it may correlate with anti-nucleic acid binding, a common characteristic of anti-nuclear antibodies. All together, this molecular evidence suggests a basis for a germline-encoded reactivity of V
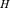
10 family members: such a cationic HCDR2 loop may bind to contiguous phosphate groups in nucleic acid-containing antigens and thus seeds a BCR/antigen interaction. Pre-BCR assembly and further BCR maturation would rely on this stimulus until it develops a new or cross-reactive specificity.

The data presented here indicate that mammals have encoded in their genome V genes sequences that are prone to bind DNA, favoring the development of anti-DNA producing B cells. However, considering the role of DNA as a driver for B-cell ontogenesis, the harmful role of anti-DNA antibodies in pathological states could be considered a trade-off phenotype, a consequence of the major role of nucleic acid in the modeling the naive repertoire. Thus, it is plausible that other autoantigens may work similarly for modeling the repertoire [4]. Therefore, large monotonous antigens from cell debris are immediately available for newly formed B-cells and immature B-cells, and may cross-link pre-BCRs, even at low affinity, to deliver survival signals that are important for B-cell development. Therefore this germline affinity may contribute to the establishment of the immature repertoire and may act secondarily in the formation of a mature, frontline repertoire.

In conclusion, we developed a experimental approach based on phage display libraries along with high throughput sequencing to test whether individual mouse V gene segments may carry an intrinsic affinity for autoantigens that promotes B-cell maturation in early ontogeny stages. Our findings indicate that intrinsic affinity for self antigens is a selected trait in animal evolution as part of the mechanism for B-cell development. This finding implies that cross-reactivity is a natural feature of unique antibodies and that the polyclonal response, as seen experimentally, is a reflex of an emergent propriety of the secondary affinities of a collection of antibodies derived from a large number of self-driven naive B-cells.

## Materials and Methods

### Database Searching

Antibody sequences were retrieved from the NCBI (National Center for Biotechnological Information) GenBank database using the BlastIg tools. All mouse rearranged heavy chain variable domain (V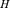) protein sequences were downloaded. Anti-nucleic acid-specific V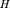 sequences were found by searching for the strings “anti-DNA”, “anti-ssDNA”, “anti-dsDNA”, or “anti-RNA” from the International Immunogenetics Information System (http://www.imgt.org). A list of accession numbers was derived from the html search file and used to retrieve the V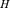 sequences from GenBank. Heavy chain variable gene fragment (V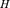) sequences were classified into families using blastp [27] against a mouse V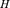 germline database based on the C57 Black mouse genome [19]. Linux shell scripts were used for most analyses.

### Phage Display

The hypothesis of an intrinsic bias toward V
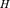
10 family binding to DNA was tested using a phage display experiment. Either a V
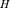
10 or V
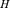
4 germline V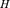 gene fragment was transferred to the pCIg 816 phagemid vector [20]. This plasmid codes for a scFv (V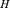 linker V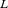) fused to the M13 gene 8 at its carboxi terminus. The germline V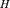 sequences were cloned so that they replaced the original V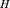 sequences. The Light chain variable domain (V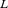) was derived from an anti-Z-DNA antibody, Z44, which uses a V
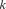
21.2 gene fragment fused to the germline J
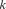
2 [21]. A synthetic CDR3 library was introduced between the V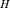 and the linker, as depicted in Figure 6. The library was introduced by amplifying the entire plasmid (reverse PCR) with one variable and one fixed HCDR3 primer. After amplification, the PCR band (4.3 Kb) was digested with *Bsi*W I (*Sun* I) and circularized with T4 DNA ligase. The circular plasmid includes a variable HCDR3 after either V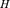 gene segment. The artificial HCDR3 is nine amino acids long, permitting the complete variability of the first six codons (NNS) and a partial restriction of codons seven to nine (KBG HTK GMT) to closely resemble the natural variability at these positions (100 g, 100 h and 100 i, kabat numbering). HCDR3 position ten was fixed to tyr, and a consensus FR4 was included (WGQGT) prior to a *Xba* I site. The HCDR3 was preceded by three codons (VRE) that include the *Bsi*W I cloning site (Figure S1).

### Library Selection

Both HCDR3 libraries (containing either V
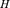
10 or V
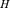
4 germline segments) were used to transform *Escherichia coli* XL1-Blue by electroporation, and fusion phage libraries were obtained using the VCSM13 helper phage (Stratagene) with standard procedures [28]. Anti-DNA specific phages were selected on oligo(dT)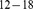 Cellulose (GE Amershan) in a pull-down experiment selection strategy. For this experiment, 500 mg of resin was resuspended in 10 mL of 10 mM Tris-HCl (pH 7.4) buffer. Before each round of selection, 50 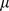
L of the resin was blocked by incubation with 500–1000 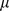
L of 3% BSA in PBS solution for one hour under agitation at room temperature. Then, phages were added (500 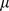
L in the first round and 50 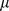
L in the other two). Following one hour of incubation under agitation at room temperature, the samples were centrifuged at 800 g for 3 minutes, and the supernatant was discarded. The unbounded phages were removed by progressive washes (5, 10 and 15) during the rounds of selection. Washes were performed by adding 1 mL of 0.05% TBST (TBS buffer with 0.05% tween 20), resuspending the resin by pipetting vigorously up and down five times, and then centrifuging as described above. After the last wash of each cycle, the resin was resuspended in 100 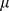
L SB broth and used directly to infect properly grown XL1-Blue *E. col*i cells. For each cycle, the input and the output phage titers were measured as described elsewhere [28].

### ss-DNA Binding Assays

To test the ssDNA binding ability of selected phages, we first carried out a dot blotting analysis. Phages of individual randomly selected clones from each round were produced by inoculating *E. coli* XL-1-Blue colonies in 1 mL of SB broth supplemented with carbenicilin (100 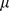
g/mL) and tetracyclin (10 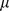
g/mL) in 96-deep-well plates. The plates were sealed, and holes were punctured in each well to facilitate aeration. Incubation was carried for 9 hours under agitation. To generate fusion phage particles, 10 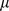
L of the helper phage was added to each well, and the culture was incubated for 90 minutes. Next, kanamycin was added to a final concentration of 70 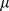
g/mL, and the plates were incubated overnight. All incubations were performed at 37°C under 300 rpm agitation. The phages were collected by centrifugation (15 min; 4°C; 4000 rpm), and 5 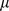
L was transferred to a nitrocellulose membrane. The membrane was blocked (5% non-fat milk in PBS) for one hour at room temperature and washed three times with 0.05% tween 20 PBS. Then, the membrane was incubated with 1 M oligo(dT)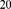-biotin solution for 2 hours at room temperature. After three washes (as described above), the membrane was incubated with alkaline phosphatase conjugated streptoavidin (Sigma, 1∶1,500 dilution) under the same conditions. The binding was revealed by adding NBT/BCIP solution. The helper phage was used as a negative control. Positive clones were selected for conventional sequencing and then for phage ELISA Assays.

To perform phage ELISA, the chosen *E. coli* clones were inoculated in 5 mL of SB broth supplemented with carbenicilin (50 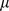
g/mL) and grown for 6 hours at 37°C under 300 rpm agitation. Fifty microliters of helper phage was added, and after 2 hours of incubation, kanamycin was supplemented to a final concentration of 70 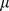
g/mL. The cultures were incubated overnight under the same conditions. After centrifugation, phages were obtained from supernatants by PEG-NaCL precipitation. Serial dilutions of phages from an initial concentration of 104 cfu/mL were transferred to a streptoavidin - oligo(dT) biotin microtiter plate. After three washes with PBST, sheep anti-M13 polyclonal antibody (Pharmacia Biotech, 1∶2,000 dilution) was added, followed by donkey anti-sheep antibody (Santa Cruz, 1∶2,000 dilution). Then, 1 mg/mL PNPP solution (para-nitro-phenyl-phosphate) was added. Absorbance at 405 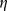
m was measured in Microplate Reader (BioRad model 450). The helper phage was used as negative control.

### Phage Sequencing and Analysis

Selected phage clones were grown in *E. coli* XL1-Blue in standard alkaline plasmids mini-preparation [29] and subject to dideoxide sequencing on a MegaBace 500 (GE) sequencer. M13 reverse and forward primers were used to analyze the HCDR3 region. The obtained sequences were analyzed with the tools PHRED and CAP3 in the platform PHPH available on the webpage www.biomol.unb.br/phph [30]. After the quality analysis, sequences were manipulated in the software BioEdit Sequence Alignment Editor [31]. Sequence alignments were performed using ClustalW [32]. PDB model 1CBV were analyzed and rendered with VMD [33].

### Deep Sequencing of Phage Libraries

To overcome the limit of Sanger sequencing for analyzing a huge number of sequences at once, we deep sequenced the original libraries and their third round of selection. We called them V
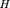
4 round zero, V
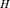
4 round three, V
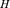
10 round zero and V
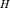
10 round three. Four forward primers were designed for each library containing an identification barcode [34], [35], and a common reverse primer was designed to amplify all libraries. The 77 bp amplicon was designed to comprise a barcode initial sequence and the complete HCDR3 sequence. Four different PCR reactions were performed to amplify each library. Equal quantities of each library, 250 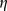
g, were gathered in one microtube, vacuum dried and sent to a high throughput sequencing facility (Scripps Institute, San Diego, CA) in a Illumina R HiSeq 2000 using the paired-end method. The resulting sequences had 2×150 bp data sets (R1 and R2) coding for complementary reads. The base identification platform was CASAVA 1.8.

### Data Analysis

The Bioinformatics pipeline developed in this work was divided into three main steps: filtering, classification and analysis. In the first step, filtering, the FASTQ sequences received from the Illumina R HiSeq 2000 were filtered by quality. In the second step, the filtered sequences were classified among the four libraries, and only the 27 nucleotide substring equivalent to the HCDR3 was maintained. The criteria for a valid HCDR3 were based on a perfect match of regular expression alignment on the edge of HCDR3 on both the V
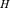
10 and V
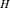
4 sequences. Those nucleotide sequences were translated, and both the nucleotide and the peptide unique sequences were counted. Sequences in the complementary R2 set were considered only when the corresponding R1 sequence was discarded due to low quality or HCDR3 string match failure. The TAG codon was translated as glutamine due to the use of a Sup44 *E. coli* strain TG1 that uses a Gln-tRNA to suppress the amber stop codon. Next, four different archives were obtained, each equivalent to one library and containing two columns, the first comprising the raw sequence and the second comprising the counted sequence. Subsequently, the counting archives were used in the final pipeline step, analysis. Enriched and depleted sequences were analyzed according to the findings of Ryvkin et al [36]. The nucleotide sequences set were used in the studies of HCDR3 composition and Kullback-Leibler divergence, while the peptide sequence sets were also used for calculating the enrichment, pattern comparison and variability. The variability study comprised an implementation of the Shannon’s entropy equation in the Perl computing language, as follows [37]:
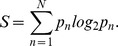




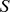
: total sequence entropy


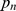
: observed 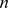 frequency


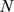
: number of distinct sequence symbols, 4 for DNA and 20 for proteins.

Information content was determined using the Weblog server (http://weblogo.berkeley.edu/) [38]. This measurement reflects the difference between the observed Entropy (S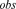) and the maximum theoretical entropy (S
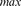
–S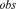). S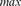 is calculated using the above formula, considering that every base or residue appears at the same frequency (0.25 for bases and 0.005 for residues) [38].

Kullback-Leibler’s divergence is calculated using the information theory and quantifies the distance between two probabilistic distributions [39]. We used it to compare the observed nucleotide and amino acid frequencies in round three using the observed frequency of round zero. The Perl programming language was used for this purpose. The formula is as follows:
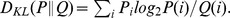




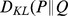
): Kullback-Leibler’s divergence


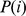
: observed distribution


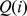
: model distribution.

## Supporting Information

Figure S1 **Detailed information about phage display vector assembly and HCDR3 library creation.**
(DOCX)

Figure S2
**Information content of HCDR3 libraries compared to the theoretical expected distribution (NNS)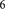 KBG HTK GMT.** Theoretical and experimental entropy was calculated using Weblogo considering nucleotide variability in the HCDR3. Nucleotide entropy (S
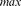
–S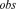) for every HCDR3 nucleotide position is shown. Upper panel display the theoretical model considering the synthetic HCDR3 linker diversity. Note that positions with N are computed as maximum entropy and are plotted with 0 by the weblogo. V
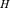
4 and V
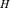
10 libraries appear below for round zero (original library) and round 3. From this comparison, it is clear that the assembly of HCDR3 suffers from bias that is most likely intrinsic to phage assembly and/or viability.(TIF)

Figure S3 **Frequency of dominant peptides changes during selection.** The frequency of dominant peptides YLLSPLLLA and VQQVNNALA occurrence in the phage pool is compared before and after four rounds of selection.
(DOCX)

Figure S4
**Molecular graphics representation of the VH10-containing antibody BV04-01 in complex with (dT)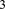.** a) The residues of V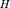 and V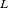 closer than 3.0 Å to the antigen (labeled NT) are detached. Residues S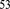 and N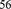 are involved in hydrogen bond with antigen’s phosphate group 1, R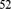 makes an ion pair with phosphate group 2. The V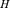 is in shown in yellow and V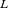 is shown in purple. b) The Van der Wall surface of the V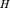 and V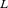 is shown in contact with the antigen. The residues R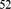, S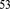 and N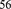 introduce a wall that contacts phosphate groups 1 and 2. c) Electrostatic surface of the variable domain interacting surface. Phosphate groups are colored gold. The first two phosphate groups are in close contact with a positively charged wall produced by R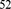 and N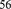. The third phosphate group is associated with positively charged surface in both the V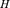 and V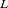 domains. d) The mature V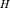 gene segment sequence is shown. Dots represent identical residues, and dashes represent gaps. The HCDR2 is the most variable region and is marked in light gray.(TIF)

Table S1 (PDF)

Table S2 **List of the 37 VH IMGT entries.**
(PDF)
