# Supplementary material for: A Mouse Variable Gene Fragment Binds to DNA Independently of the BCR Context: A Possible Role for Immature B-Cell Repertoire Establishment
Source: PLoS One. 2013 Sep 2;8(9):e72625. doi: 10.1371/journal.pone.0072625 (PMC3759382; doi:10.1371/journal.pone.0072625)
Supplement: Figure S1 — Detailed information about phage display vector assembly and HCDR3 library creation. (DOCX) [file pone.0072625.s001.docx]

**Supplementary Figure 1**

**Oligonucleotide NNS:**

**Fw 3 CDR H3 Fw4**

**Seq.** 5´C GTA CGA GAA NNS NNS NNS NNS NNS NNS KBG HTK GMT TAT TGG GGT CAA GGA ACC T 3´

*Bsi*W I

**Aa:**  Val Ala Glu Xxx Xxx Xxx Xxx Xxx Xxx Ala Met Asp Tyr Trp Gly Gln Gly Thr

Gly Leu Ala

Val Phe

Ser

Trp

Leu

## Complement Oligonucleotide

Seq: 5´GCG CTC TAG AGG AAA CGG TGA CTG AGG CTT GAC CCC AA 3

*Xba* I ´

**Oligonucleotides used to generate the CDR3H library.** To generate the CDR3H variant template, oligonucleotide NNS was designed presenting six complete degenerated codons (NNS) and three partial degenerated ones (KBG, HTK and GMT). This oligonucleotide also show a *Bsi*W I restriction site. A complementary oligonucleotide was also designed. The CDR3H variants were obtained annealing these two oligonucleotides and proceeding a DNA polymerase Klenow fragment reaction. The amplicons were double digested with *Bsi*W I *and Xba* I and purified for cloning steps despicted below. Legend: N = A, C, G or T; S= A or T; K= G or T; B= C, G or T; H= A, C or T; M= A or C; and Xxx= any amino acid. The conserved immunoglobulin WGXG motif at the base of FW4 is highlited in red.

**
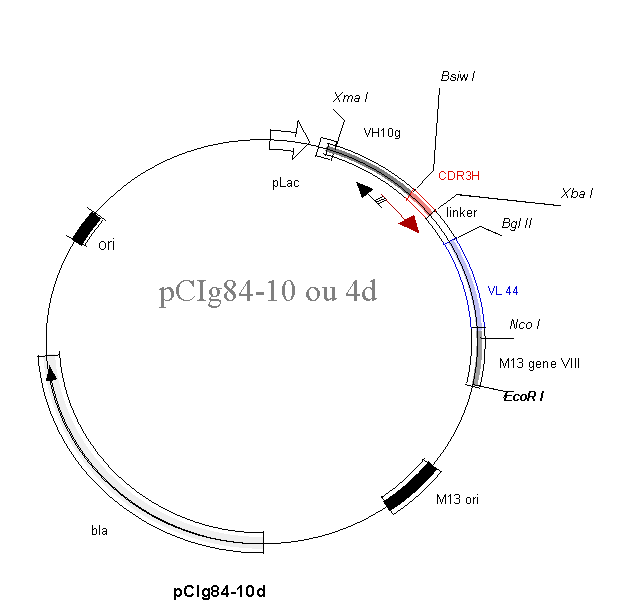
**

**pCIg 8410 or pCIg844**

4,3 kb

**Construction of pCIG 8410 and pCIg 844 vectors and generation of CDR3H libraries.** The plasmids used as template for generation of CR3H libraries were constructed from pCIg 816 vector (Maranhão & Brígido, 2000). First genomic DNA from C57/Black6 mouse hepatocytes was used to obtain VH10 germline sequence. A nested PCR amplification was accomplished using primers 5’ AGGCAGTTGCGGACAATATA 3’ (forward) and 5’ *CGTACG*CAGTAATACATGGCTGTGT 3’ (reverse) in a first reaction, and then the purified amplicon was used as template for a second reaction, replacing the forward primer for 5’ *CCCGGG*AGGTGCAGCTTGTTGAG 3’. The final PCR product was cloned into pGEM T vector (Promega) and presented *Xma* I and *Bsi*W I restriction sites in 5´and 3´ ends, respectively (showed in italics in the nested primers sequences). To obtain pCIg 8410, the cloned amplicon was purified after a double digestion with *Xma* I and *Bsi*W I and ligated with both, pCIg 816 digested with *Xma* I and *Xba* I and the *Bsi*W I and *Xba* I digested CDR3H variant obtained as described above. The triple ligation was performed using T4 DNA Ligase (New England Biolabs), according with manufactures´ instructions. As a final step Z44 VL coding gene was cloned in the new vector, between Bgl II and Nco I sites, replacing the Z22 VL. The final vector, pCIg 8410 was used for generation of CDR3H library in VH10 context. pCIG 844 was obtained replacing VH10 germiline coding sequence by VH4 germiline one. This germiline segment was obtained from Balb/c mouse liver genomic DNA amplification in nested PCR using for the first step the oligonucleotides 5´ CAACACTGAACACACATCC 3´ (foward) and 5´CTTCCTTGTTTCTCATTTCC 3´ (reverse). For the secondo step 5´ *CCCGGG*AGGTGAAGCTTCTCGAGTC 3´ (forward) and 5´ T*CGTACG*CAGTAATAAAGGGCTGTTG 3´ (reverse). The 300pb final PCR product harbored *Xma* I and *Bsi*W I sites used for cloning steps. Both vectors, pCIg 8410 and pCIg 844 were used as template in inverse PCR reactions, aiming the generation of CDR3H libraries. In this PCR amplification the whole circular plasmids are used as template. For both plasmids the CDR3H libraries were generated using the common oligonucleotide 5´ TATTACTG*CGTACG*AGAANNSNNSNNSNNSNNSNNSKBGHTKGMTTATTGG 3´ and the specific ones 5´ TTCT*CGTACG*CAGTAATAAAGGGCTGTGTCCTCAGATCTCACTT 3´ and 5´ TTCTCGTACGCAGTAATACATGGCTGTGTCCTCAGTTTTCAGGT 3´, for VH4 and VH10, respectively. The amplification was accomplished using the GeneAmp XL kit (Perkin Elmer), following manufacturers´ recommendation. Briefly, after an initial denaturation step (93°C, 1 minute), 30 cycles of amplification were performed (94°C 1 min. followed by f5min and 30 seconds extension). After a final extension step (72°C, 10 minutes), the linear product was cleaved with *Bsi*W I, eluted from agarose gel and ligated prior to transform electrocompetent XL1-blue *E. coli* cells. .
