# Supplementary figures and images for: A Mouse Variable Gene Fragment Binds to DNA Independently of the BCR Context: A Possible Role for Immature B-Cell Repertoire Establishment
Source: PLoS One. 2013 Sep 2;8(9):e72625. doi: 10.1371/journal.pone.0072625 (PMC3759382; doi:10.1371/journal.pone.0072625)

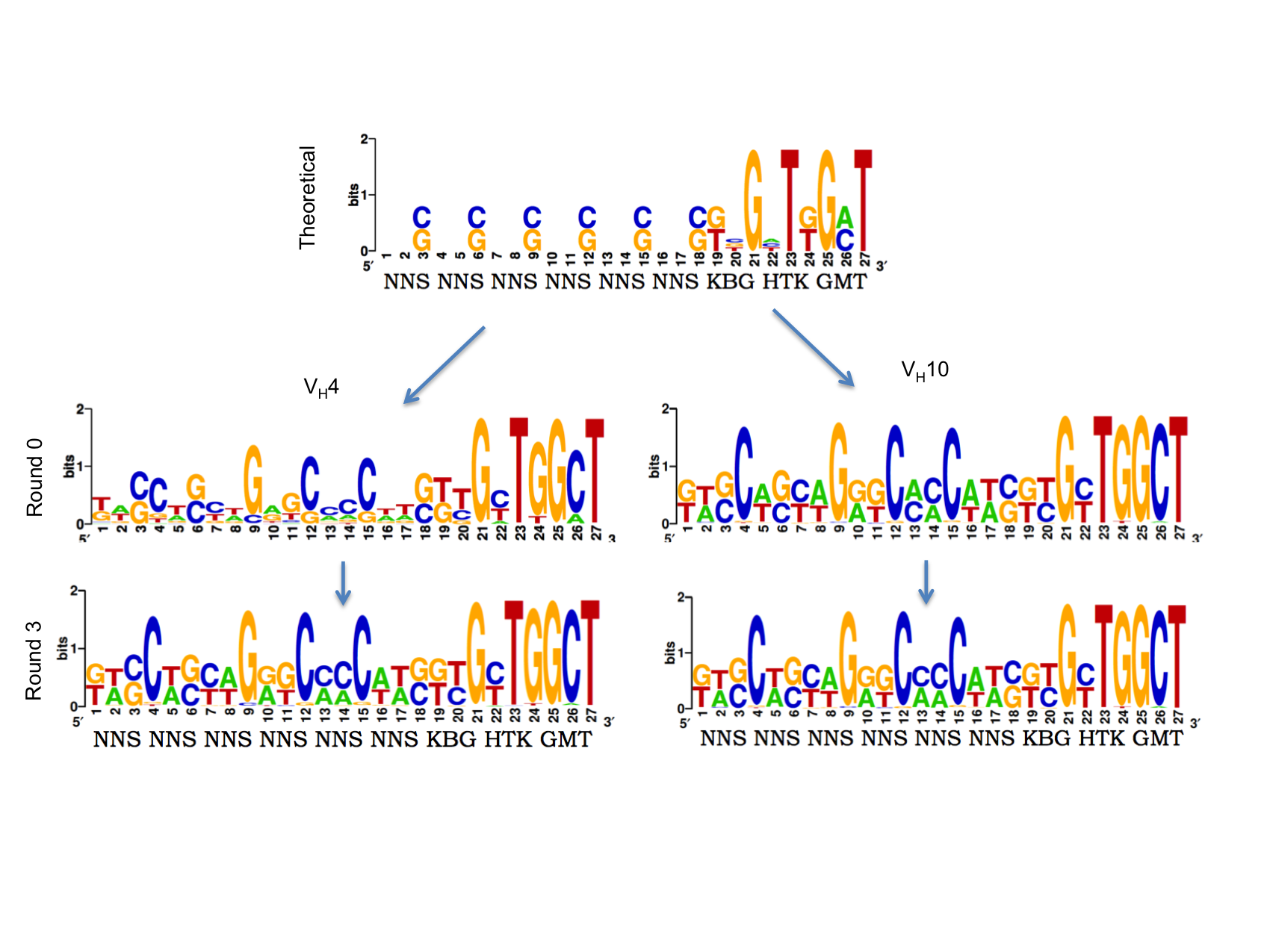

Supplement: Figure S2 — Information content of HCDR3 libraries compared to the theoretical expected distribution (NNS) KBG HTK GMT. Theoretical and experimental entropy was calculated using Weblogo considering nucleotide variability in the HCDR3. Nucleotide entropy (S–S) for every HCDR3 nucleotide position is shown. Upper panel display the theoretical model considering the synthetic HCDR3 linker diversity. Note that positions with N are computed as maximum entropy and are plotted with 0 by the weblogo. V4 and V10 libraries appear below for round zero (original library) and round 3. From this comparison, it is clear that the assembly of HCDR3 suffers from bias that is most likely intrinsic to phage assembly and/or viability. (TIF) [file pone.0072625.s002.tif]

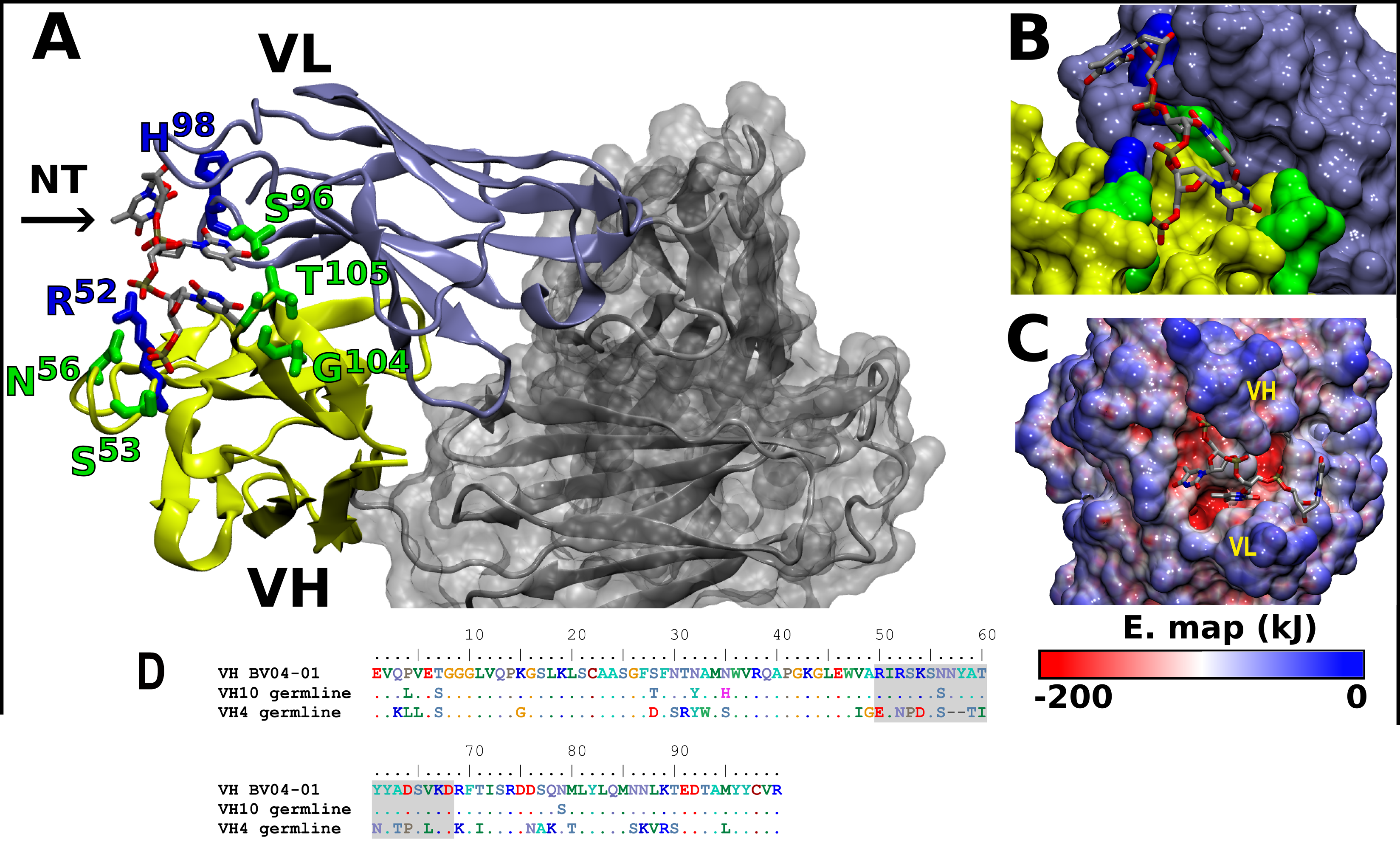

Supplement: Figure S4 — Molecular graphics representation of the VH10-containing antibody BV04-01 in complex with (dT). a) The residues of V and V closer than 3.0 Å to the antigen (labeled NT) are detached. Residues S and N are involved in hydrogen bond with antigen’s phosphate group 1, R makes an ion pair with phosphate group 2. The V is in shown in yellow and V is shown in purple. b) The Van der Wall surface of the V and V is shown in contact with the antigen. The residues R, S and N introduce a wall that contacts phosphate groups 1 and 2. c) Electrostatic surface of the variable domain interacting surface. Phosphate groups are colored gold. The first two phosphate groups are in close contact with a positively charged wall produced by R and N. The third phosphate group is associated with positively charged surface in both the V and V domains. d) The mature V gene segment sequence is shown. Dots represent identical residues, and dashes represent gaps. The HCDR2 is the most variable region and is marked in light gray. (TIF) [file pone.0072625.s004.tif]
