# Supplementary material for: A Mouse Variable Gene Fragment Binds to DNA Independently of the BCR Context: A Possible Role for Immature B-Cell Repertoire Establishment
Source: PLoS One. 2013 Sep 2;8(9):e72625. doi: 10.1371/journal.pone.0072625 (PMC3759382; doi:10.1371/journal.pone.0072625)
Supplement: Figure S3 — Frequency of dominant peptides changes during selection. The frequency of dominant peptides YLLSPLLLA and VQQVNNALA occurrence in the phage pool is compared before and after four rounds of selection. (DOCX) [file pone.0072625.s003.docx]

**Supplementary Figure 3. Dominant peptides were also selected in oligo-dT**

**S3. Frequency of dominant peptides changes during selection.** The frequency of YLLSPLLLA and VQQVNNALA occurrence in the phage pool is compared before and after three rounds of selection.
