# Supplementary material for: A Mouse Variable Gene Fragment Binds to DNA Independently of the BCR Context: A Possible Role for Immature B-Cell Repertoire Establishment
Source: PLoS One. 2013 Sep 2;8(9):e72625. doi: 10.1371/journal.pone.0072625 (PMC3759382; doi:10.1371/journal.pone.0072625)
Supplement: Table S1 — (PDF) [file pone.0072625.s005.pdf]

Supplementary Table 1

VH Family	Prototype	sequences Found[a]	Frequency of Family[b]	Number of Germline Sequences[c]	Frequency of Family / Number of Germlines	Nucleic Acid Binding Sequences[d]	Frequency of ligands in Family[e]	Frequency of Ligands / Number of Germlines[f]
VH1	J558	2,348	66.6	52	1.28	422	18.0	0.35
VH2	Q52	237	6.7	9	0.75	96	40.5	4.50
VH3	36-60	148	4.2	6	0.70	19	12.8	2.14
VH4	X-24	32	0.9	1	0.91	0	0.0	0.00
VH5	7183	327	9.3	10	0.93	120	36.7	3.67
VH6	J606	101	2.9	5	0.57	14	13.9	2.77
VH7	S107	170	4.8	3	1.61	45	26.5	8.82
VH8	3609	44	1.2	8	0.16	5	11.4	1.42
VH9	VGAM3-8	66	1.9	4	0.47	6	9.1	2.27
VH10	DNA4	37	1.1	2	0.53	22	59.5	29.73
VH11	CP3	9	0.3	2	0.13	0	0.0	0.00
VH14	SM7	4	0.1	1	0.11	0	0.0	0.00
TOTAL		3,523	100.0	103	0.97	749		

[a]Number of VH sequences in database
[b]Fraction of sequences of each VH Family
[c]as describe for C57/BL6 (Johnston et al., 2006)
[d]Number of sequences described as anti-DNA
[e]Ratio of nucleic acid binding to the total sequence in Family
[f]Ratio of nucleic acid binding in Family to the number of family's germlines
