# Supplementary material for: A Mouse Variable Gene Fragment Binds to DNA Independently of the BCR Context: A Possible Role for Immature B-Cell Repertoire Establishment
Source: PLoS One. 2013 Sep 2;8(9):e72625. doi: 10.1371/journal.pone.0072625 (PMC3759382; doi:10.1371/journal.pone.0072625)
Supplement: Table S2 — List of the 37 VH IMGT entries. (PDF) [file pone.0072625.s006.pdf]

List of the 37 VH IMGT entries

anti-DNA VH10			not described as anti-DNA VH10		
GI	ID	Annotation	GI	ID	Annotation
90696	I32513	Ig heavy chain precursor V region (MRL4)	285179	B42575	Ig heavy chain V region (anti-angiotensin)
194549	AAA51055.1	immunoglobulin heavy chain	2253316	AAB62898.1	IgMk heavy chain variable region
194551	AAA51056.1	immunoglobulin heavy chain	2253340	AAB62910.1	IgMk heavy chain variable region
196952	AAA38849.1	IgG2ak heavy chain precursor	2570571	AAB82170.1	anti-fluorescein monoclonal IgM heavy
284849	PH1091	Ig heavy chain V region (clone P17.79)	2570573	AAB82171.1	anti-fluorescein monoclonal IgM heavy
284850	PH1092	Ig heavy chain V region (clone P17.80)	5853172	AAD54347.1	immunoglobulin heavy chain variable*
284851	PH1093	Ig heavy chain V region (clone S17.161)	5853180	AAD54351.1	immunoglobulin heavy chain variable*
284962	A41940	Ig heavy chain V region G2b, autoantibody	5853182	AAD54352.1	immunoglobulin heavy chain variable*
288686	CAA80054.1	immunoglobulin variable region	29374080	AAO73015.1	anti-meningococcal polysaccharide
288740	CAA80056.1	immunoglobulin variable region	52858386	AAU89119.1	anti-preS1 immunoglobulin heavy
288836	CAA80067.1	immunoglobulin variable region	61814564	AAX56286.1	anti-lipoteichoic acid heavy chain
493918	1CBV	H Chain H, An Autoantibody To	61814570	AAX56288.1	anti-lipoteichoic acid heavy chain
494400	1NBV	H Chain H, An Autoantibody To	61814580	AAX56290.1	anti-lipoteichoic acid heavy chain
1872251	AAB49064.1	anti-DNA immunoglobulin heavy chain	61814592	AAX56292.1	anti-lipoteichoic acid heavy chain
1872253	AAB49065.1	anti-DNA immunoglobulin heavy chain			
1872279	AAB49078.1	anti-DNA immunoglobulin heavy chain			
1872361	AAB49119.1	anti-DNA immunoglobulin heavy chain			
1872409	AAB49143.1	anti-DNA immunoglobulin heavy chain			
1872411	AAB49144.1	anti-DNA immunoglobulin heavy chain			
1872413	AAB49145.1	anti-DNA immunoglobulin heavy chain			
2906050	AAC04511.1	anti-poly(dC) monoclonal antibody			
3309229	AAC26038.1	anti-DNA immunoglobulin IgM heavy			
3309239	AAC26043.1	anti-DNA immunoglobulin IgM heavy			

Repited entree (shaded rows: 90696, 284849, 284850, 284851, 493918, 494400)

*Cross-reactive with DNA
